# ID1 promotes hepatocellular carcinoma proliferation and confers chemoresistance to oxaliplatin by activating pentose phosphate pathway

**DOI:** 10.1186/s13046-017-0637-7

**Published:** 2017-11-23

**Authors:** Xin Yin, Bei Tang, Jing-Huan Li, Yan Wang, Lan Zhang, Xiao-Ying Xie, Bo-Heng Zhang, Shuang-Jian Qiu, Wei-Zhong Wu, Zheng-Gang Ren

**Affiliations:** 1 Liver Cancer Institute & Zhong Shan Hospital, Fudan University, 136 Yi Xue Yuan Road, Shanghai, 200032 China; 2 Key Laboratory of Carcinogenesis and Cancer Invasion, Ministry of Education, Shanghai, China

**Keywords:** Hepatocellular carcinoma, ID1 (inhibitor of differentiation and DNA binding-1), Pentose phosphate pathway, Chemoresistance

## Abstract

**Background:**

Drug resistance is one of the major concerns in the treatment of hepatocellular carcinoma (HCC). The aim of the present study was to determine whether aberrant high expression of the inhibitor of differentiation 1(ID1) confers oxaliplatin-resistance to HCC by activating the pentose phosphate pathway (PPP).

**Methods:**

Aberrant high expression of ID1 was detected in two oxaliplatin-resistant cell lines MHCC97H–OXA(97H–OXA) and Hep3B–OXA(3B–OXA). The lentiviral shRNA or control shRNA was introduced into the two oxaliplatin-resistant cell lines. The effects of ID1 on cell proliferation, apoptosis and chemoresistance were evaluated in vitro and vivo. The molecular signaling mechanism underlying the induction of HCC proliferation and oxaliplatin resistance by ID1 was explored. The prognostic value of ID1/G6PD signaling in HCC patients was assessed using the Cancer Genome Atlas (TCGA) database.

**Results:**

ID1 was upregulated in oxaliplaitin-resistant HCC cells and promoted HCC cell proliferation and oxaliplatin resistance. Silencing ID1 expression in oxaliplaitin-resistant HCC cell lines inhibited cell proliferation and sensitized oxaliplaitin-resistant cells to death. ID1 knockdown significantly decreased the expression of glucose-6-phosphate dehydrogenase (G6PD), a key enzyme of the PPP. Silencing ID1 expression blocked the activation of G6PD, decreased the production of PPP NADPH, and augmented reactive oxygen and species (ROS), thus inducing cell apoptosis. Study of the molecular mechanism showed that ID1 induced G6PD promoter transcription and activated PPP through Wnt/β-catenin/c-MYC signaling. In addition, ID1/G6PD signaling predicted unfavorable prognosis of HCC patients on the basis of TCGA.

**Conclusions:**

Our study provided the first evidence that ID1 conferred oxaliplatin resistance in HCC by activating the PPP. This newly defined pathway may have important implications in the research and development of new more effective anti-cancer drugs.

**Electronic supplementary material:**

The online version of this article (10.1186/s13046-017-0637-7) contains supplementary material, which is available to authorized users.

## Background

Hepatocellular carcinoma (HCC) is one of the most common cancers worldwide and the major cause of cancer-related death [1]. Only about 20% patients with HCC are candidates for surgical resection [2]. In most cases, the disease has progressed to an intermediate or advanced stage at the time of diagnosis. Transcatheter arterial chemoembolization (TACE) or systemic chemotherapy may improve the survival of patients with advanced HCC [3], but acquired drug resistance remains an obstacle in further improving the postoperative outcome of HCC patients.

Oxaliplatin, a third-generation platinum analogue, is a compound with significant anti-cancer activities against colorectal, breast, gastric, renal carcinomas and sarcomas [4]. It also has been employed in combination with 5-fluorouracil (5-FU) and leucovorin as the first-line chemotherapy regimen (FOLFOX4) for advanced HCC [5]. As a bifunctional alkylating agent, oxaliplatin can covalently bind DNA and form platinum-DNA adducts that block DNA replication and transcription [6]. However, ample evidence has shown that the occurrence of chemoresistance is a major limitation to the efficacy of platinum-based therapies in managing HCC [7, 8]. Molecular mechanisms involved in oxaliplatin resistance of HCC remain poorly defined.

ID1, an inhibitor of differentiation and DNA binding-1 and a member of the helix-loop-helix (HLH) transcription factor family [9], has been known to play a crucial role in mammary epithelial cells and cancer cells by mediating diverse cellular functions, including inhibition of differentiation, delaying replicative senescence, promotion of cell proliferation, invasion and metastasis [10]. Clinically, a high ID1 level is positively associated with a poor patient outcome. For instance, the prognosis was reported to be poor in early-stage cervical cancer patients with enhanced ID1 expression [11]. Increased ID1 expression in breast cancer patients was associated with more aggressive behavior and shorter overall survival (OS) [12]. In patients with non small-cell lung cancer (NSCLC), high ID1 expression was associated with poor survival and resistance to chemotherapy or radiotherapy [13]. However, few data are currently available regarding the role of ID1 in promoting chemoresistance in HCC. The result of gene expression profiling analysis in our previous study showed that ID1 was highly expressed in oxaliplatin-treated HCC tumors, and maintained stem cell characteristics through increasing autocrine of insulin-like growth factor 1 (IGF1) [14]. In the present study, we found that high expression of ID1 promoted HCC cell proliferation and conferred oxaliplatin resistance to HCC by regulating G6PD, a key enzyme of pentose phosphate pathway, which may represent a novel target to improve the therapeutic efficacy of patients with advanced HCC.

## Methods

### Cell culture and establishment of oxaliplatin-resistant HCC cell lines

MHCC97H (97H), a high-metastatic human HCC cell line, was established in Liver Cancer Institute of Zhongshan Hospital [15]. MHCC97H cells from the 12th to the 15th passage were used in our experiments. Hep3B, a low metastatic potential HCC cell line was purchased from the America Type Culture Collection(ATCC, HB 8064™). The oxaliplatin-resistant HCC cell lines MHCC97H–OXA and Hep3B–OXA selected at a 25umol/L concentration of oxaliplatin were successfully established from MHCC97H and Hep3B, by exposing cells to gradually increasing oxaliplatin (Sigma, St. Louis, MO, USA) from 2 umol/L to 25umol/L in our laboratory [14]. The IC50 value of surviving HCC cells treated with oxaliplatin was about 10-fold as high as that of their parental cells (Additional file 1: Figure S1).MHCC97H and MHCC97H–OXA were cultured in Dulbecco’s Modified Eagle’s Medium (DMEM, Gibco Invitrogen, Carlsbad, CA, USA) supplemented with 10% fetal bovine serum (FBS, Life Technologies/Gibco). Hep3B and Hep3B–OXA cells were cultured in minimum essential medium (MEM) supplemented with 10% fetal bovine serum. Cells(1 × 10^6^) were seeded into 25 cm culture flask for 72 h per passage.

### Gene knockdown and rescue

**Fig. 1:**
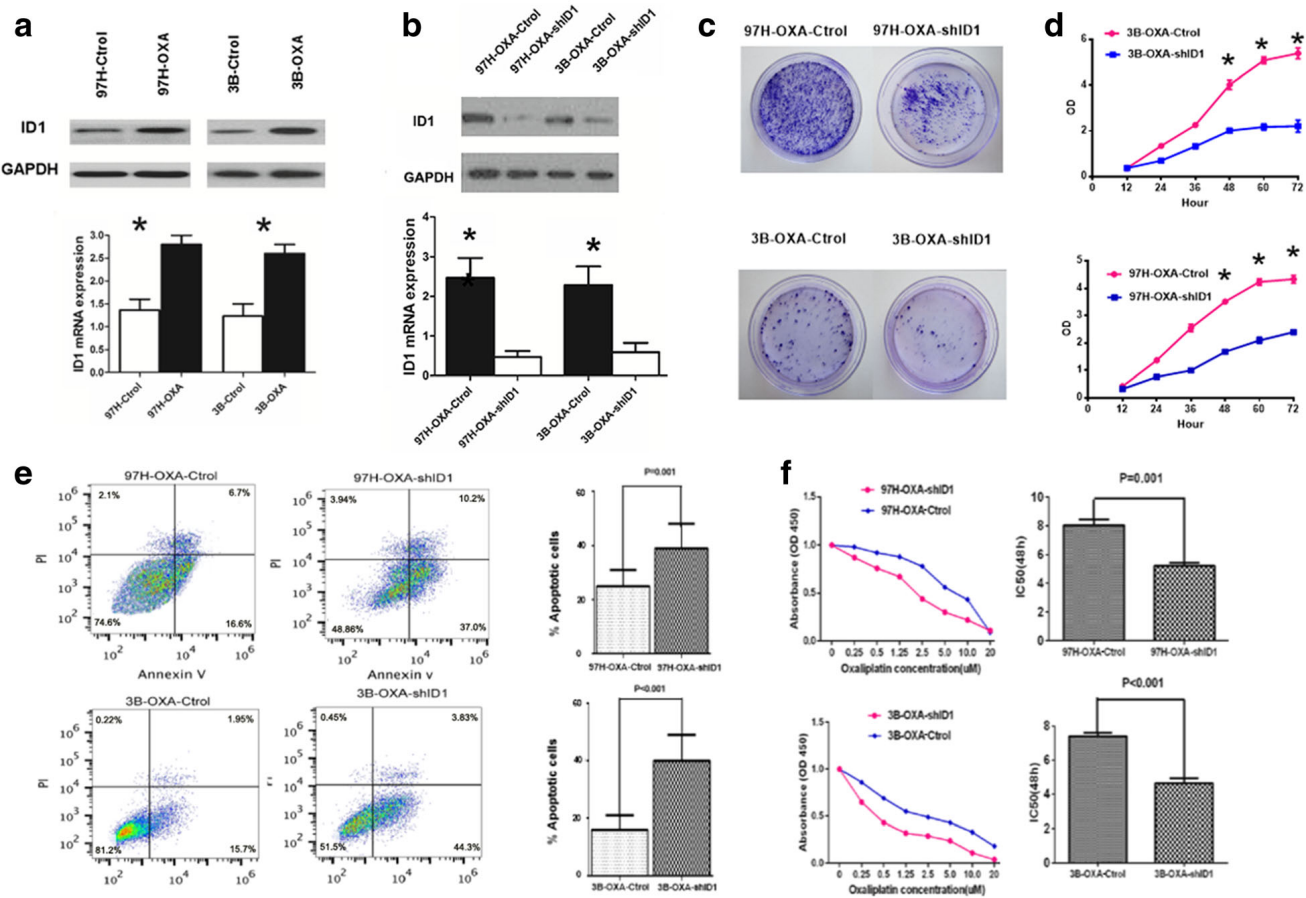
ID1 is overexpressed in oxaliplaitin-resistant HCC cell lines and involved in HCC cell proliferation, apoptosis and chemoresistance in vitro. **a** ID1 expression was analyzed by Western blot and Real-time RT-PCR assays in oxaliplaitin-resistant HCC cell lines. **b** 97H–OXA and 3B–OXA cells were infected by lentiviral vectors with short hairpin RNA clone targeting ID1 gene. After transfection, ID1 knockdown was confirmed by western blot analysis and Real-time RT-PCR assays. **c** 97H–OXA-Ctrol, 97H–OXA-shID1, 3B–OXA-Ctrol and 3B–OXA-shID1 cells were subjected to colony formation ability assay. **d** CCK-8 assay showed that the proliferation of 97H–OXA-shID1 and 3B–OXA-shID1 cells was inhibited upon silencing ID1 expression (* *P* < 0.05). The data are indicated as means of three independent assays. **e** Cell apoptosis was determined by flow cytometry using Annexin V FITC apoptosis detection kit. The number of apoptotic 97H–OXA-shID1 and 3B–OXA-shID1 cells was significantly increased upon silencing ID1 expression (*P* = 0.001 and *P* < 0.001). **f** After treatment with different concentrations of oxaliplaitin for 48 h, the chemoresistance of HCC cells was evaluated by CCK-8 assay. The IC50 value in 97H–OXA or 3B–OXA cells was significantly higher than that in 97H–OXA-shID1 or 3B–OXA-shID1 cells (*P* = 0.001 and *P* < 0.001, respectively)

For gene knockdown, short hairpin RNA (shRNA) clones targeting ID1 were delivered by lentiviral infection.shRNA targeting sequences for ID1 genes are 5′-gatccAACTCGGAATCCGAAGTTGGATTCAAGAGATCCAACTTCGGATTCCGAGTTTTTTTTg-3′.5 × 10^4^ cells were seeded in 6-well plates 24 h before addition of lentivirus and 8 μg/mL polybrene to the growth medium, and then cultured for additional 72 h. Three days After transfection, ID1 knockdown was confirmed by Western blot analysis and qPCR (Fig. 1b).For rescue experiments, 2 μg G6PD plasmid was transfected into ID1-knockdown 97H–OXA and 3B–OXA cells for 48 h using Lipofectamine 2000 (Life Technologies, Carlsbad, CA, USA).

### PCR Array study

Human Cancer Pathway Finder™ PCR Array that profiles the expression of 84 genes representative of 9 different biological pathways (listed in Table 1) was purchased from SABiosciences (Qiagen Company, Milano, Italy). Total RNA isolated from 97H–Ctrol and 97H–shID1 cells was used for screening by real-time PCR according to the manufacturer’s instruction. Target genes whose expressions were differentially regulated (with at least 2-fold difference) were selected and validated using real-time PCR and Western blot.

**Table 1:** Gene expression profiling after ID1 silenced in MHCC97H cells based on PCR array

Functional gene groups	Symbol	Uni.Gene	RefSeq	Descrption	Fold Change
Metabolism	ACLY	Hs.387567	NM_001096	ATP citrate lyase	3.03
	G6PD	Hs.461047	NM_000402	Glucose-6-phosphate dehydrogenase	−9.80
Apoptosis	BIRC3	Hs.127799	NM_001165	Baculoviral IAP repeat containing 3	3.88
	CASP2	Hs.368982	NM_032982	Caspase 2, apoptosis-related cysteine peptidase	−2.22
Hypoxia	CA9	Hs.63287	NM_001216	Carbonic anhydrase IX	8.44
	EPO	Hs.2303	NM_000799	Erythropoietin	2.30
	HMOX1	Hs.517581	NM_002133	Heme oxygenase (decycling) 1	−2.94
Cell Senescence	IGFBP3	Hs.450230	NM_000598	nsulin-like growth factor binding protein 3	2.81
	IGFBP5	Hs.607212	NM_000599	Insulin-like growth factor binding protein 5	−2.56
	SERPINB2	Hs.594481	NM_002575	Serpin peptidase inhibitor, clade B (ovalbumin), member 2	4.69
Cell Senescence	TBX2	Hs.531085	NM_005994	T-box 2	−2.50
Angiogenesis	SERPINF1	Hs.532768	NM_002615	Serpin peptidase inhibitor, clade F (alpha-2 antiplasmin, pigment epithelium derived factor), member 1	2.33

### Real-time PCR and western blot

Total RNA was isolated using Trizol reagent (Invitrogen, Carlsbad, CA) and reverse-transcribed to cDNA using PrimeScript RT reagent Kit (DRR037A, Takara, Japan). For real-time PCR (RT-PCR), SYBR Premix Ex Taq (DRR081, Takara, Japan) was used according to the manufacturer’s instructions. The qPCR primers used are provided in Additional file 2: Table S1. Western blots were performed as described previously [16]. Images were captured through Gel Doc XR system (Bio-Rad, Philadelphia, PA), and analyzed using Image LabTM software (version 2.0). Antibodies against ID1 and c-MYC were brought from Santa Cruz Biotechnology (Santa Cruz Biotechnology, CA, USA). Antibodies against G6PD, β-catenin, TCF-4 and Lamin B were brought from Abcam (AbCam, Cambridge, UK). Antibodies against Caspase-3 and Caspase-9, and Ki67, ERK, and pERK antibodies were obtained from Cell Signaling Technology (Cell Signaling Technology, Danvers, MA). Antibody against GAPDH was from Kangcheng Technology (Shanghai, China).

### CCK-8 assay and colony formation ability assay

CCK-8 assay was carried out as previously described [17]. Briefly, 100 μL cells (2 × 10^3^/ml) were seeded in triplicated wells of a 96-well microplate. After 5-day culture, 10 μL CCK-8 solution (Beyotime, Haimen, China) was added to each well and incubated at 37 °C for 2 h. Absorbance values were expressed as percentages relative to the controls.

For colony formation ability assay, cells were plated in 60 mm^2^ culture dishes at 2 × 10^3^ cells/well for 7–14 days. Colonies were stained with Giemsa (Beyotime, Haimen, China), and surviving colonies (a colony was defined as >50 cells) were counted.

### Flow cytometry analysis

Cell apoptosis was determined by flow cytometry using FITC Annexin V Apoptosis Detection Kit (BD Pharmingen, San Diego, CA, USA). Briefly, 1 × 10^6^ cells were washed with ice-cold phosphate-buffered saline (PBS) twice and resuspended in 200 μl 1 × binding buffer containing 2.5 μl Annexin V-PE for 15 min at room temperature in the dark. After incubation, the population of Annexin V-positive cells was evaluated using a FACS Aria cytometer (Becton cytomics FC500, Fremont, CA, USA).

### G6PD enzyme activity

The enzyme activity of G6PD was assessed using a G6PD assay kit (Sigma, St. Louis, MO, USA), according to the manufacturer’s instructions. The combined activity of G6PD and 6-phosphogluconate dehydrogenase (6PGD), the second enzyme of the PPP that also produces NADPH, was determined by the rate of conversion of NADP+ to NADPH in the presence of glucose-6-phosphate (G6P). G6PD activity was calculated by subtracting 6PGD from the combined activity. The reaction buffer contained 50 mM Tris (pH 8.1) and 1 mM MgCl_2_, and the substrate concentration was G6P(200 uM), 6-phosphogluconate(6PG,200 μM) and NADP+ (100 μM). Enzyme activities were normalized based on the protein concentration, which was determined by a Bio-Rad protein assay kit (Bio-Rad, Richmond, CA, USA).

### NADPH and ROS levels

NADPH levels and NADP+/NADPH ratios were determined using the NADP+/NADPH Quantification kit (BioVision, Mountain View, CA, USA). Intracellular reactive oxygen species (ROS) generation was measured by using Cell ROX Deep Red reagent (Life Technologies Carlsbad, CA, USA) according to manufacturer’s protocol. Analysis and data acquisition were performed in Beckman cytomics FC500 by using CellQuest Pro (BD) analysis software (FACS analysis).

### Coimmunoprecipitation

Coimunoprecipitation was performed according to the manufacture’s protocol (Cell Signaling, Frankfurt, Germany). Briefly, 5 × 10^5^ cells were lysed in lysis buffer (100 mM TRIS/HCl pH 7.5, 150 mM NaCl, 1 mM MgCl_2_, 0.25% NP-40, 5 mM NaF, 1 mM Na_3_VO_4_, 10 μg/mL Pepstatin, 100 μM PMSF and 3 μg/mL Aprotinin) and incubated with Rabbit serum (DAKO, Glostrup, Denmark)) for 1 h on ice and Agarose A/G-protein bead (Santa Cruz Biotechnology, CA, USA) for 30 min. The supernatant was immunoprecipitated with ID1 antibody (1:500) overnight at 4 °C. Immune complexes were precipitated with Agarose A/G-protein bead for 2 h at 4 °C min and analyzed by immunoblotting. Comparable results were obtained in at least two-independent experiments.

### Dual luciferase assay

Cells growing to 70% confluence were transfected in triplicate with pGL3-G6PD-luc, pcDNA c-MYC or pGL3-Basic (Promega, Madison, WI, USA), along with pRL-TK. After 48-h transfection, cells were collected and the luciferase activity was measured using the Dual-Luciferase Reporter Assay System (Promega, Madison, WI) according to the manufacturer’s protocol. The luciferase activity of pRL-TK served as internal control.

### Animal treatment

Male athymic BALB/c nude mice (Shanghai Institute of Material Medicine of the Chinese Academy of Sciences) were raised in specific pathogen-free conditions. Animal care and experimental protocols were conducted in accordance with guidelines established by the Shanghai Medical Experimental Animal Care Commission. Cells (5 × 10^6^ cells per mouse) were injected subcutaneously into the upper left flank region of each mouse to produce tumors. Seven days later, mice were treated with 0.1 ml oxaliplatin (10 mg/kg/week) via tail vein injection for 4 weeks. The excised tumors were fixed with 4% formaldehyde and embedded in paraffin. The expression of Ki-67, Caspase-3 and Caspase-9 was detected using immunohistochemistry, and apoptotic cells in tumors were detected by TUNEL (terminal deoxynucleotidyl transferase dUTP nick end labeling) according to the manufacturer’s instructions (Boyetime, Shanghai, China). The percentage of TUNEL-positive cells to the total cell number was recorded as an apoptotic index.

### Transcription factor binding sites analysis and TCGA database analysis

Jaspar (http://jaspardev.genereg.net), an open-access database of the transcription factors binding preferences in multiple species, was used to predict potential transcription factor binding sites. Survival analysis data were obtained from TCGA database using the UCSC Cancer Genomics Browser (https://genome-cancer.ucsc.edu). A total of 371 HCC samples were collected in the dataset. The relationship between ID1/G6PD and the prognosis of HCC patients was explored.

### Statistical analysis

All experiments were repeated at least three times and representative results are presented. All values in the figures and text are the means ± SD. Statistical analyses were performed using the SPSS 13.0 for Windows (SPSS, Inc., Chicago, IL). Any significant difference between mean values was evaluated by Student t test or Mann-Whitney U test. A two-sided *P* < 0.05 was accepted as significant.

## Results

### ID1 is up-regulated in oxaliplaitin-resistant HCC cell lines and involved in HCC cell malignant proliferation, apoptosis and chemoresistance in vitro

It was found in our previous study that oxaliplaitin-resistant HCC cells acquired stem cell-like traits, and gene profile analysis revealed 267 up-regulated and 65 down-regulated genes in oxaliplatin-treated HCC tumors, compared with GS-treated HCC tumors [14]. These differentially expressed genes were related to chemokines, chemokine receptors, signal transduction, inflammation, proliferation, development, and metabolism. Among them, ID1 was highly expressed in oxaliplatin-treated HCC tumors. Therefore, we hypothesized that ID1 was probably activated in chemoresistant HCC cells. To confirm this finding, the expression of ID1 in oxaliplaitin-resistant HCC cells was examined. As expected, the expression level of ID1 in 97H–OXA and Hep3B–OXA cells was significantly higher than that in their parental cells (Fig. 1a). We further established 97H–OXA-shID1 and 3B–OXA-shID1 cells transfected with ID1shRNA to down-regulate endogenous ID1 expression (Fig. 1b). Colony formation ability assay (Fig. 1c) and CCK-8 assay analysis (Fig. 1d) revealed that ID1 knockdown inhibited the proliferation of HCC cells markedly in the absence of oxaliplaitin (*P* < 0.05). In addition, silencing ID1 expression induced the apoptosis MHCC97H–OXA and 3B–OXA cells (Fig. 1e). We then investigated whether ID1 was also involved in HCC chemoresistance. After 48-h treatment with a series of diluted concentrations of oxaliplatin, the half-maximal inhibitory concentration (IC50) of oxaliplaitin in ID1 knockdown 97H–OXA-shID1 and 3B–OXA-shID1 cells was significantly lower than that in control cells (Fig. 1f). These data demonstrated that ID1 was involved in the regulation of proliferation, apoptosis and chemoresistance in oxaliplaitin-resistant HCC cells.

### Silencing ID1 expression inhibits HCC cell malignant proliferation, apoptosis and chemoresistance in vivo

**Fig. 2:**
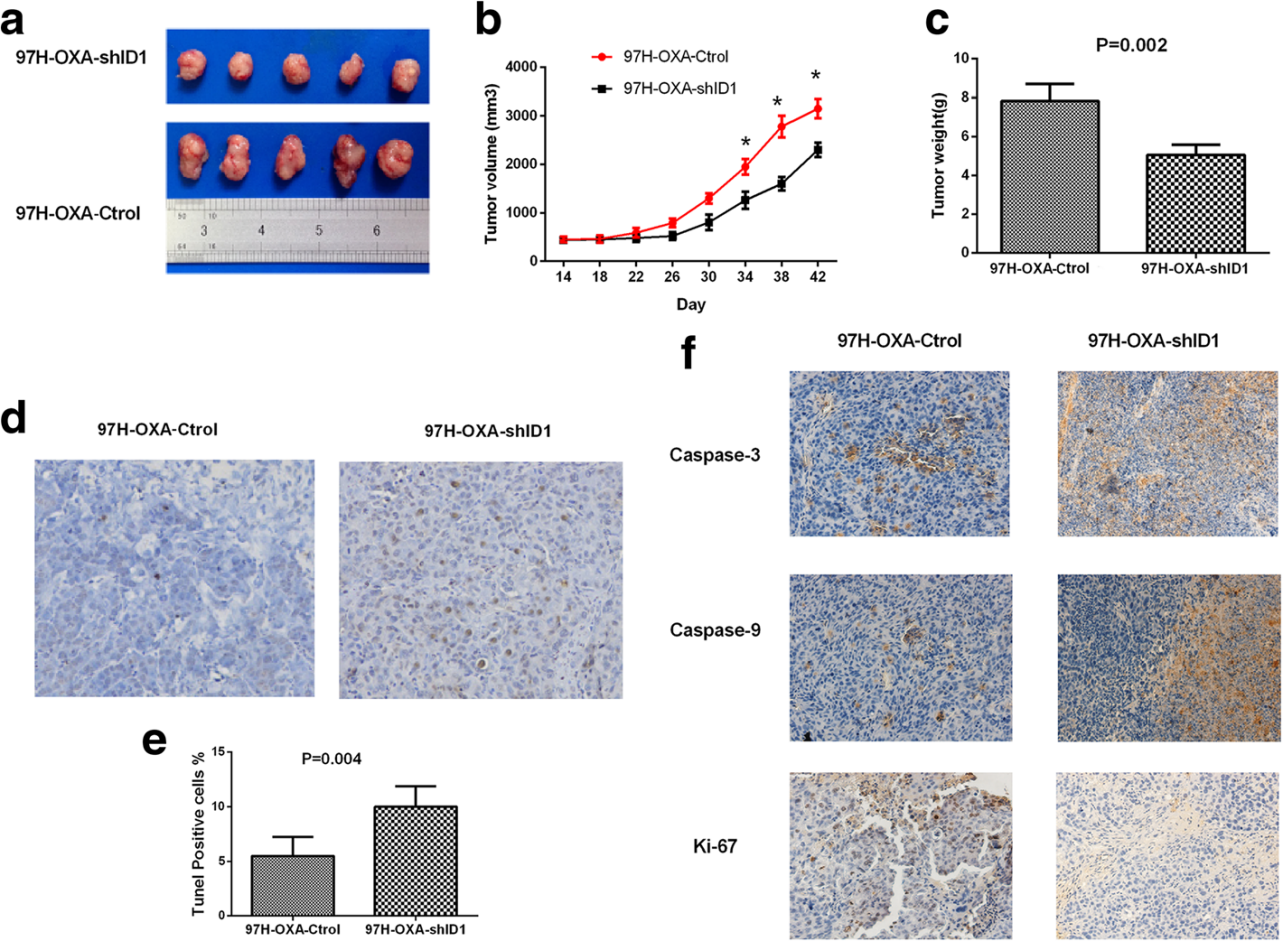
Silencing ID1 expression inhibits HCC cell proliferation, apoptosis and chemoresistance in vivo. **a** 97H–OXA-Ctrol and 97H–OXA-shID1 cells were injected subcutaneously into the right flank of nude mice. One week after implantation, mice were treated with 0.1 ml oxaliplatin (10 mg/kg/week) via tail vein injection for 4 weeks. **b** The graph of tumor kinetics showed that ID1 knockdown inhibited tumor growth in 97H–OXA-shID1 group treated with oxaliplatin (* P < 0.05) (**c**) 6 weeks after implantation, 97H–OXA-Ctrol cells produced larger tumors than 97H–OXA-shID1 cells (*P* = 0.002). The data are presented as the mean ± SD of five tumors per group. **d** and **e** The apoptosis rate in 97H–OXA-shID1 cells was higher than that in 97H–OXA-Ctrol cells as determined by TUNEL analysis (P = 0.002). **f** IHC analysis of the apoptosis-related and proliferation-related protein expression in 97H–OXA-Ctrol tumor and 97H–OXA-shID1 tumor. ID1 knockdown increased the expression of Caspase-3 and Caspase-9 and inhibited the expression of Ki-67

Based on the vitro findings described above, we examined the impact of ID1 on HCC malignant proliferation, apoptosis and chemoresistance in vivo. Xenografts in nude mice were established by subcutaneous injection of 97H–OXA-Ctrol cells and 97 L–OXA-shID1 cells into nude mice as described in the Methods section. Seven days after implantation, nude mice were treated with 0.1 ml oxaliplatin (10 mg/kg/week) via tail vein injection for 4 weeks. In all cases, the mean size of tumors formed by 97H–OXA-Ctrol group was significantly larger than that in 97H–OXA-shID1 group (*P* < 0.05) (Fig. 2a and b). In addition, the final mean tumor weight in 97H–OXA-Ctrol group was significantly higher than that in 97H–OXA-shID1 group (*P* = 0.002) (Fig. 2c), indicating that ID1 knockdown inhibited tumor proliferation of 97H–OXA cells in vivo. We then examined in vivo apoptosis of the xenograft, using TUNEL staining. Conversely with the tumor size, tumors derived from 97H–OXA-shID1 cells displayed a higher percentage of TUNEL-stained cells compared with the tumor cells (Fig. 2d and e). Furthermore, we detected the expression levels of Caspase-3, Caspase-9 and Ki-67 by immunohistochemistry and found that the expressions of Caspase-3 and Caspase-9 were significantly increased in 97H–OXA-shID1 tumors compared with 97H–OXA-Ctrol tumors (Fig. 2f). In addition, the expression of Ki-67 in 97H–OXA-shID1 group was significantly decreased compared with that in 97H–OXA-Ctrol group. These results indicate that knockdown of ID1 expression inhibited cell proliferation, promoted cell apoptosis, and chemosensitized oxaliplaitin-resistant HCC cells to oxaliplaitin in vivo.

### Glucose-6-phosphate dehydrogenase (G6PD), a key enzyme of pentose phosphate pathway, is significantly down-regulated when ID1 is silenced in HCC cells

**Fig. 3:**
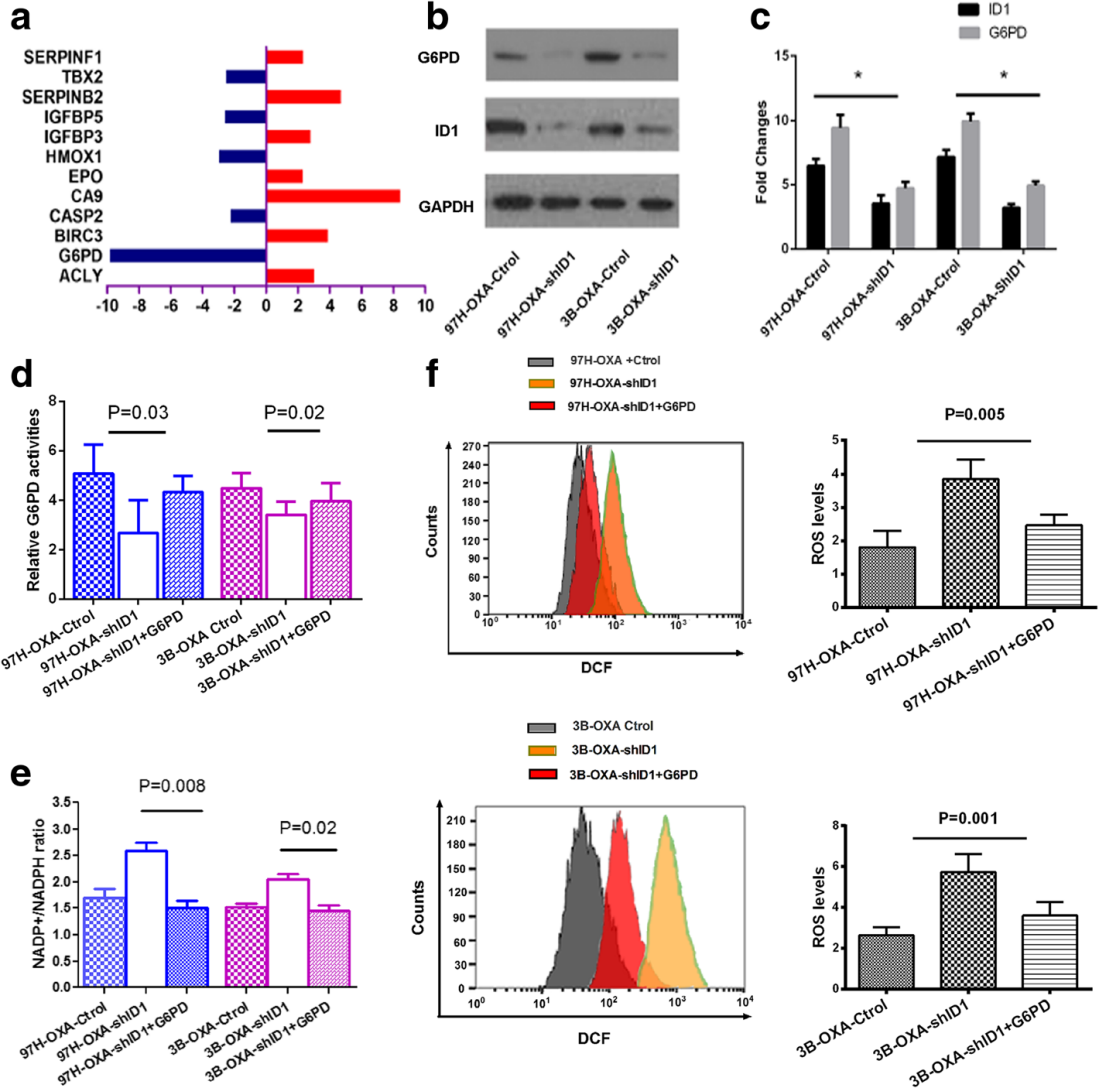
ID1 activates pentose phosphate pathway and confers chemoresistance to oxaliplatin in HCC through G6PD. **a** PCR array analysis of the differentially expressed genes in 97H Ctrol and 97H–shID1 cells. **b** and **c** Among these differentially expressed genes, G6PD was downregulated upon ID1 knockdown, as illustrated by Western blot and Real-time RT-PCR assays(* P < 0.05). **d** The enzyme activity of G6PD was measured in oxaliplaitin resistant cell lines. ID1 knockdown inhibited the enzyme activity of G6PD in 97H–OXA-shID1 and 3B–OXA-shID1cell lines (*P* = 0.03 and *P* = 0.02). After transfecting G6PD plasmid into ID1-knockdown 97H–OXA-shID1 or 3B–OXA-shID1 cells, enzyme activity of G6PD were recovered (**e**) NADP+ /NADPH ratios were determined through enzymatic assays. ID1 knockdown inhibited NADPH accumulation. Recovery of G6PD expression in 97H–OXA-shID1 and 3B–OXA-shID1cell lines increased NADPH accumulation. Data are presented as mean ± SD of *n* = 3 independent experiments. **f** Quantification of ROS level was measured using flow cytometry assay. ID1 silencing increased ROS generation in 97H–OXA-shID1 and 3B–OXA-shID1 cell lines (*P* = 0.005 and *P* = 0.001, respectively). Recovery of G6PD expression in 97H–OXA-shID1 and 3B–OXA-shID1cell lines inhibited ROS generation. Data are presented as mean ± SD of n = 3 independent experiments

To quantitatively examine the mechanism of ID1 on cell proliferation and oxaliplaitin resistance, 84 genes related to cell proliferation, apoptosis, cell cycle, angiogenesis, invasion, and metastasis were evaluated via Human Cancer Pathway Finder™ PCR Array in 97H–Ctrol cell line and 97H–shID1 cell line. After knockdown of ID1, 12 genes exhibited significant changes in mRNA expression relative to control (expression ratio showing greater than 2.0-fold or less than 0.5-fold difference compared with the control group). Among the differentially expressed genes, 7 genes were up-regulated and 5 genes were down-regulated (Table 1 and Fig. 3a). Further real-time PCR and Western blot verified that ID1 knockdown significantly decreased the expression of G6PD, a key rate-limiting enzyme in pentose phosphate pathway (Fig. 3b and c).

### ID1 activates pentose phosphate pathway and confers chemoresistance to oxaliplatin in HCC cell lines via regulation of G6PD

**Fig. 4:**
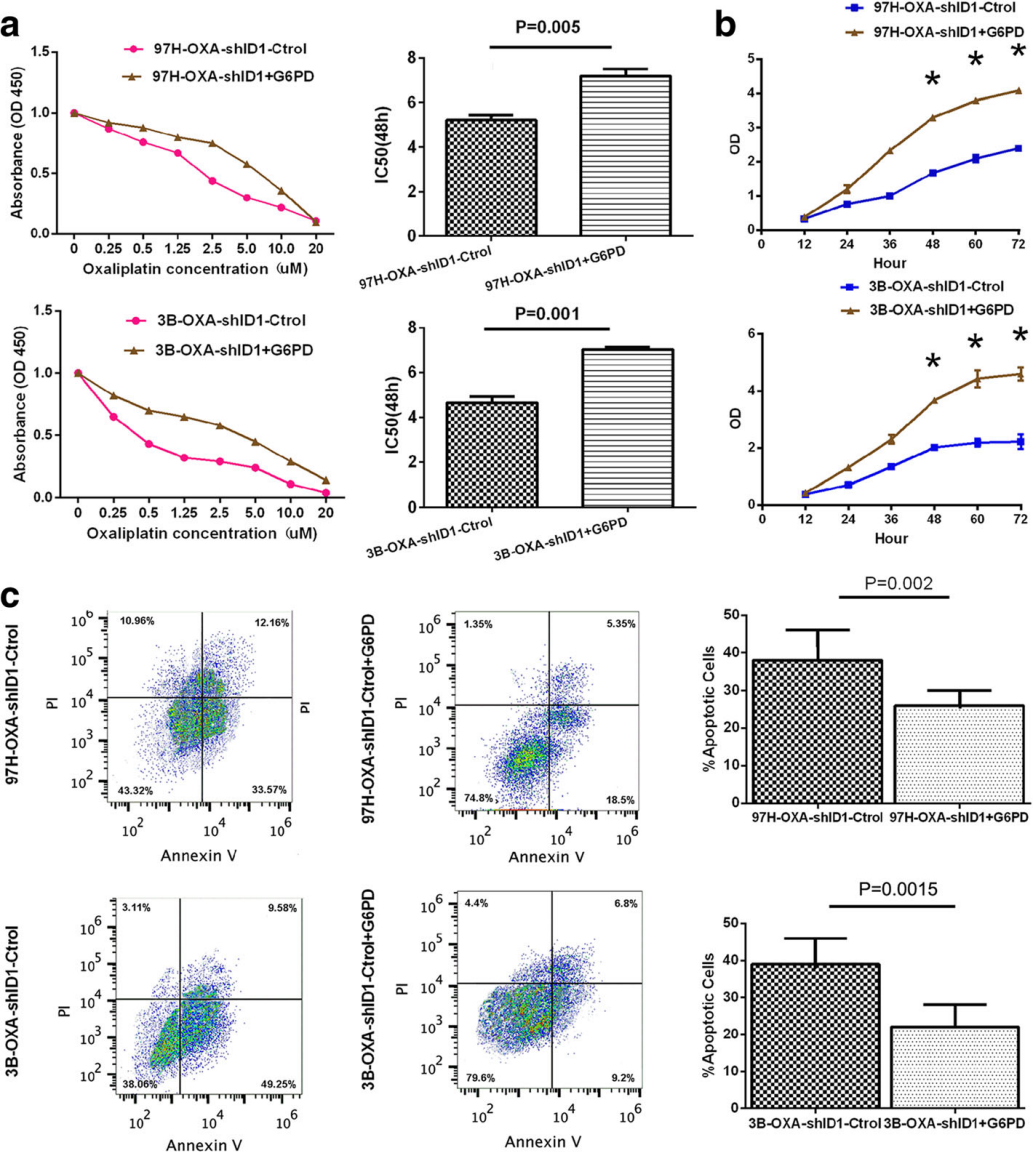
G6PD rescues HCC oxaliplatin chemoresistance by promoting HCC cell proliferation and inhibits apoptosis in spite of ID1 knockdown. **a** The oxaliplatin chemoresistance of HCC cells was evaluated by CCK-8 assay. After transfection with G6PD plasmid, the IC50 value in 97H–OXA-shID1 + G6PD cells or 3B–OXA-shID1 + G6PD cells was increased, as compared with their control cells (*P* = 0.005 and P = 0.001, respectively). **b** CCK-8 assay showed that cell proliferation of 97H–OXA-shID1 and 3B–OXA-shID1 was recovered upon G6PD transfection (* *P* < 0.05). **c** Cell apoptosis was determined by flow cytometry. The number of apoptotic 97H–OXA-shID1 + G6PD and 3B–OXA-shID1 + G6PD cells was decreased compared with their control cells (*P* = 0.002 and *P* = 0.0015)

Knowing that the key enzyme of pentose phosphate pathway G6PD may be a downstream target of ID1, we wondered whether ID1 induced-oxaliplatin resistance was mediated by the activation of pentose phosphate pathway. Thus, the enzyme activity of G6PD, the metabolic product of pentose phosphate pathway NADPH and the levels of intracellular reactive oxygen species (ROS) were further assayed in 97H–OXA-Ctrol cells and 97H–OXA-shID1cells. Compared with 97H–OXA-shID1 cells, the 97H–OXA-Ctrol cells exhibited a higher activity of G6PD (*P* = 0.003) (Fig. 3d) with a significant increase in NADPH levels (*P* = 0.008) (Fig. 3e) and a significant decrease in intracellular ROS level (*P* = 0.005) (Fig. 3f). The same results were also obtained in 3B–OXA-Ctrol cell line and 3B–OXA-shID1 cell line (Fig. 3d-f). Furthermore, after transfecting G6PD plasmid into ID1-knockdown 97H–OXA-shID1 or 3B–OXA-shID1 cells, the enzyme activity of G6PD was recovered (Fig. 3d) with an elevated NADPH level (Fig. 3e) and a decreased intracellular ROS level (Fig. 3f). More importantly, the recovery of G6PD expression seemed to induce oxaliplatin chemoresistance (P = 0.005 and *P* = 0.001) (Fig. 4a) in 97H–OXA-shID1 or 3B–OXA-shID1 cells via promoting cell proliferation (Fig. 4b) and inhibit cell apoptosis (Fig. 4c). These results indicate that ID1 promoted malignant proliferation and conferred oxaliplatin chemoresistance to HCC cells by activating pentose phosphate pathway through its downstream target G6PD.

### ID1-induced G6PD overexpression is dependent on c-MYC activation

**Fig. 5:**
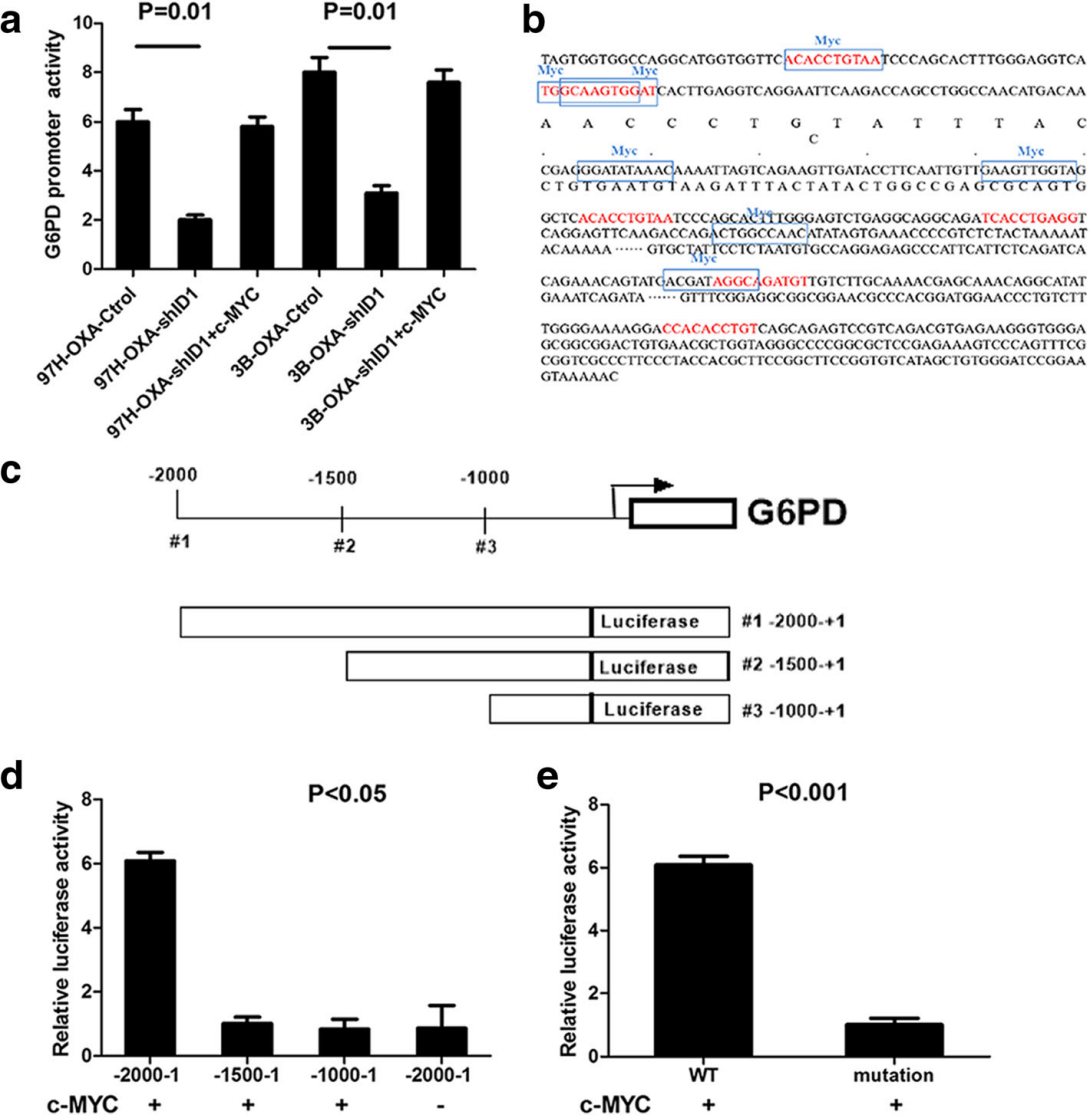
ID1 regulates G6PD transcription through c-MYC activation. **a** G6PD transcriptional activity was measured by dual luciferase reporter assay. Relative luciferase activity was normalized to the luciferase activity of control vector. As indicated, transcriptional activity of G6PD promoter was downregulated by silencing ID1 expression and was recovered upon c-MYC plasmid expression (*P* = 0.01). **b** Illustration of potential c-MYC binding sites on G6PD promoter, predicted by bioinformatic analysis. **c** Schematic representation of luciferase reporter constructs #1(−2000, +1), #2 (−1500, +1) and #3 (−1000, +1). **d** Dual luciferase assay of HEK293T cells co-transfected with plasmids containing different G6PD promoter reporter constructs with or without c-MYC plasmid. **e** Dual luciferase reporter assay of HEK293T cells co-transfected with pGL3-G6PD-WT or pGL3-G6PD-Mut (GGATATAAAC) constructs, together with c-MYC expression plasmid. Results shown are mean ± SD of three experiments

Knowing that G6PD mRNA expression in ID1-knockdown cells was reduced significantly as compared with that in control cells (Fig. 3c), suggesting that ID1 regulated G6PD at the transcriptional level. Next, by using dual luciferase assay, we investigated whether ID1 could regulate G6PD transcriptional activity. As shown in Fig. 5a, knockdown of ID1 expressions in 97H–OXA and 3B–OXA cell lines inhibited the ID1 G6PD promoter activity (*P* < 0.05). Due to the lack of the basic DNA binding region, ID1 protein usually interacts with the basic-helix-loop-helix (bHLH) transcription factor by forming heterodimers in regulating its downstream gene expression. To decipher the molecular mechanism by which ID1 regulates G6PD, we screened for bHLH transcription factors (Additional file 3: Table S2) that could possibly bind G6PD promoter by using bioinformatic analysis (http://jaspardev.genereg.net/). The result showed that the promoter sequence of G6PD harbored 7 potential c-MYC binding sites (Fig. 5b), indicating that c-MYC may be involved in ID1-induced G6PD promoter activation. Further luciferase reporter assay confirmed that after transfecting c-MYC plasmid, G6PD promoter activity was restored devoid of ID1 in 97H–OXA-shID1 and 3B–OXA-shID1 cells (Fig. 5a). This result indicates that the activating effect of ID1 on the G6PD promoter was dependent on the endogenous expression of c-MYC. To experimentally confirm whether c-MYC directly targets G6PD promoter, we transfected different lengths of G6PD promoter reporter vectors with or without c-MYC plasmids in 293 T cells. The constructs used for reporter assays are illustrated in Fig. 5C. Luciferase reporter assay showed that G6PD promoter activity was low without transfecting c-MYC plasmid (Fig. 5d). After co-transfecting c-MYC plasmid, a remarkable increase in luciferase activity was observed from full-length G6PD promoter reporter constructs, but not from the reporter vector containing −1500 bps to +1 bps promoter fragment or −1000 bps to +1 bps promoter fragment, suggesting that −2000 to −1500 region on G6PD promoter was responsible for c-MYC-mediated transcriptional activation (Fig. 5d). This region contains one c-MYC potential binding position (GGATATAAAC) on the G6PD promoter. Mutation of this position markedly reduced the G6PD promoter activity induced by c-MYC (Fig. 5e). These results strongly imply that the regulatory effect of ID1 on G6PD promoter activity was mediated by c-MYC expression.

### ID1 regulates c-MYC activation through Wnt/β-catenin pathway

**Fig. 6:**
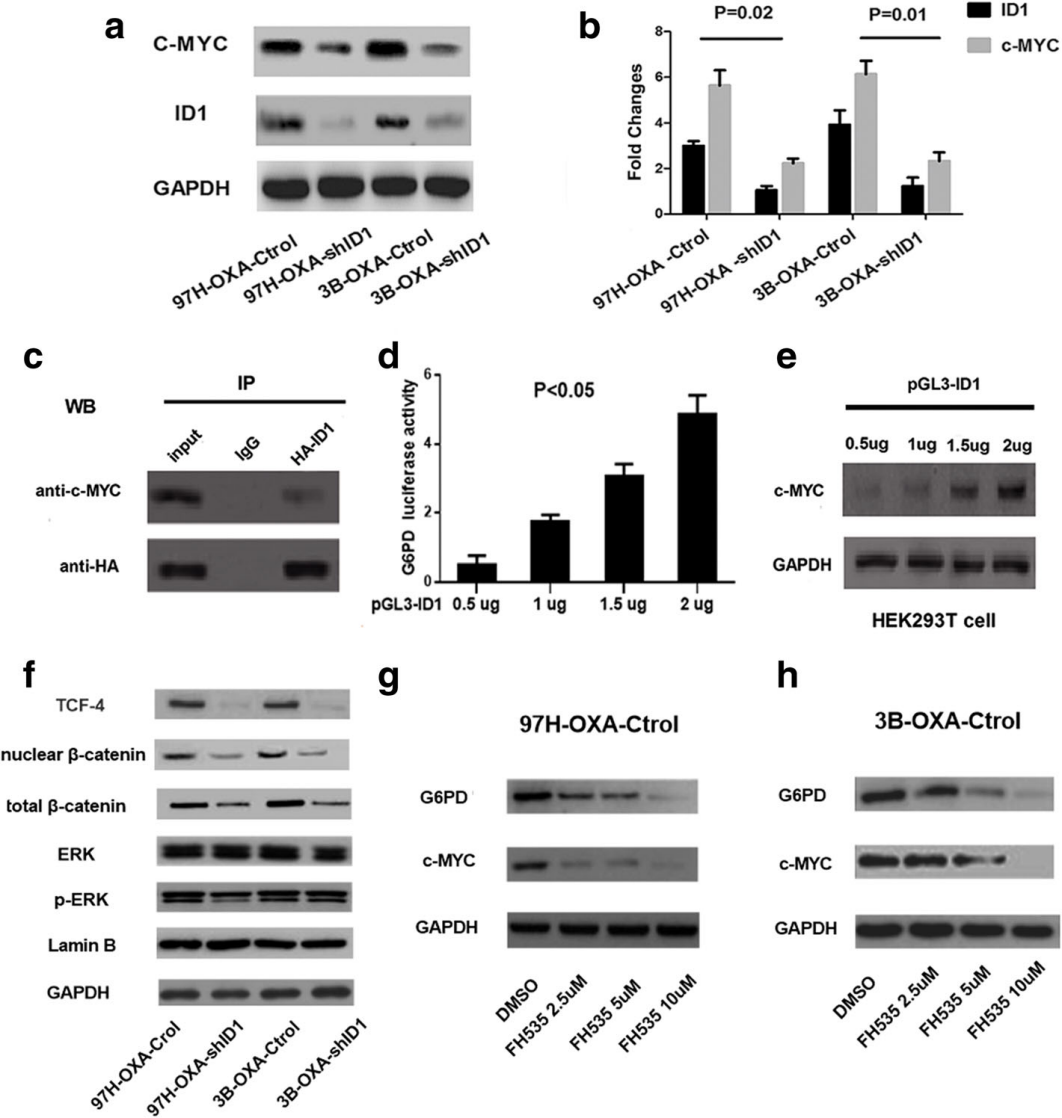
ID1 modulates c-MYC activation through Wnt/β-catenin pathway. **a** and **b** c-MYC expression was down-regulated upon ID1 knockdown, as indicated in Western blot and Real-time RT-PCR assays. **c** Direct interaction of ID1 and c-MYC protein was explored by coimmunoprecipitation. Protein was immunoprecipitated (IP) with anti-ID1 antibody and was immunoblotted to detect c-MYC expression. Normal mouse IgG was used as a control antibody. **d** G6PD promoter transcription activity was increased upon the pGL3-ID1 plasmid concentration increasing from 0.5 to 2μg. **e** Increased c-MYC expression from 0.5 to 2μg was detected in HEK293T cells transfected with pGL3-ID1 plasmid. **f** Western Blot showed that Wnt/β-catenin pathway, rather than MAPK/ERK pathway was regulated by ID1expression. **g** and **h** ID1 induced c-MYC and G6PD expression was inhibited by FH535, a specific small-molecule inhibitor of Wnt/β-catenin pathway (10umol/L for 48H)

Next, we asked whether c-MYC expression was regulated by ID1. As seen in Fig. 6a and b, c-MYC mRNA and protein expressions were reduced when ID1 expression was suppressed in 97H–OXA-shID1 cells or 3B–OXA-shID1 cells. ID proteins belong to class V of HLH factors that lack the basic domain; they dimerise with E proteins and prevent their DNA interaction, thus, acting as dominant negative of E proteins [18, 19]. Since c-MYC is a bHLH protein, it is entirely possible that ID1 directly interacts with c-MYC, controlling and affecting G6PD transcriptional activity. By coimmunoprecipitation, we detected a measurable amount of ID1/c-MYC complexes (Fig. 6c). However, strikingly, ID1 binding to c-MYC did not appear to have negative effects on the transcriptional activity of G6PD. In luciferase reporter analyses, G6PD transactivation was gradually enhanced when the pGL3-ID1 plasmid concentration was increased from 0.5 to 2 μg in the same transfection reaction (Fig. 6d), due to the argument of c-MYC expression (Fig. 6e). These results indicate that although ID1 could directly bind to c-MYC, it did not suppress its downstream gene G6PD transcriptional activity.

**Fig. 7:**
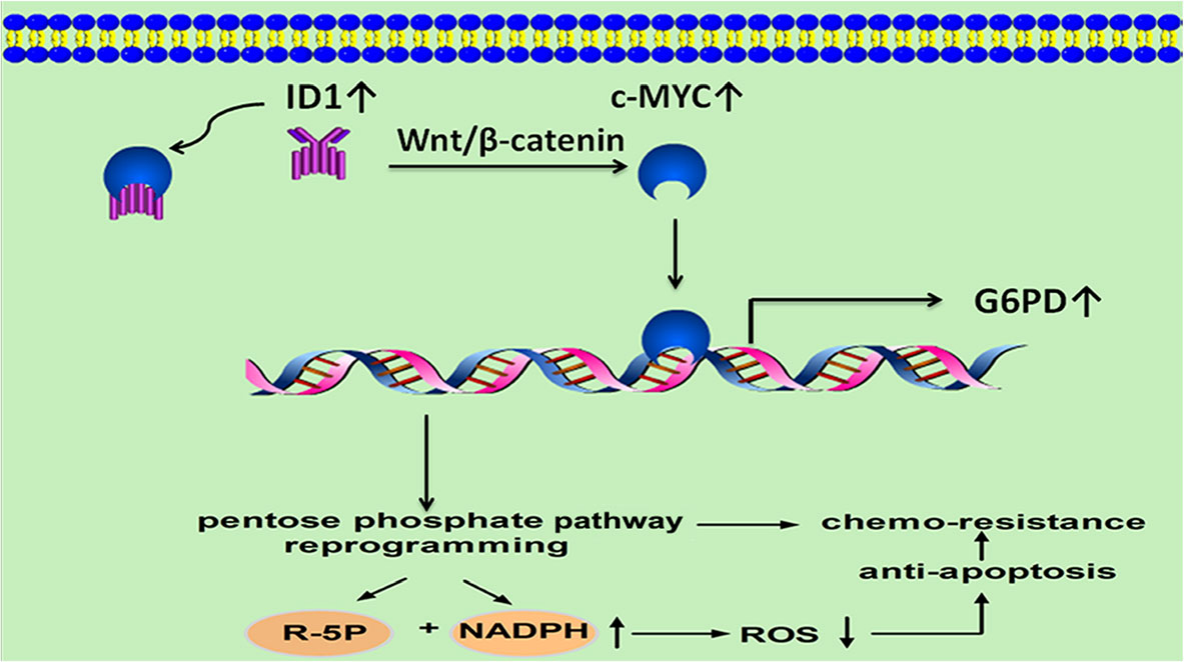
Schematic illustration of the proposed molecular mechanism of ID1-induced PPP activation and chemoresistance in HCC. ID1 regulated c-MYC through Wnt/ β-catenin pathway activation, which in turn promoted G6PD promoter transcription and activated the PPP. Activation of the PPP provided more NADPH production and downregulated intracellular ROS levels, thus encouraging chemoresistance in HCC

Knowing that ID1 regulated self-renewal of both normal and cancer stem cells via Wnt/β-catenin dependent c-MYC transcription activation [20], and ID1 promoted c-MYC expression through MAPK/ERK signal pathway in HCC [21], we next explored whether ID1 enhanced c-MYC activities through these two pathways. As is illustrated in Fig. 6f, Wnt/β-catenin pathway, rather than MAPK/ERK pathway, was activated in ID1 over-expression 97H–OXA and 3B–OXA cell lines. Down-regulation of ID1 expression inhibited Wnt/β-catenin pathway activation in 97H–OXA-shID1 cells and 3B–OXA-shID1 cells. Treatment with FH535 (Sigma-Aldrich, St. Louis, MO, USA), a small-molecule inhibitor of Wnt/β-catenin, repressed ID1-mediated c-MYC/G6PD expression in 97H–OXA and 3B–OXA cell lines (Fig. 6g and h). Together, these results indicate that ID1 induced c-MYC activation through Wnt/β-catenin pathway, thus promoting G6PD transcription, which in turn activated pentose phosphate pathway and conferred chemoresistance to oxaliplatin in HCC (Fig. 7).

### ID1/G6PD signaling predicts unfavourable prognosis in HCC

Knowing that ID1/G6PD signaling promoted tumor cell proliferation and chemoresistance in HCC, we wondered whether ID1/G6PD signaling could predict unfavourable prognosis in HCC patients. To prove this postulation, we downloaded a gene expression dataset from TCGA database using the UCSC Cancer Genomics Browser (https://genome-cancer.ucsc.edu). HCC patients were stratified as ID1/G6PD high or ID1/G6PD low based on the median values of ID1 and G6PD expression. Survival analysis indicated that the expressions of ID1 and G6PD were associated with a poor survival outcome in HCC patients (Log rank *P* = 0.001 and *P* < 0.001) (Additional file 4: Figure S2A and 2B). Moreover, the most unfavourable prognosis was observed in patients with coexpression of ID1 and G6PD among the entire cohort (Log rank P < 0.001) (Additional file 4: Figure S2C).These results demonstrate that ID1/G6PD signaling could predict a poor clinical outcome in HCC patients.

## Discussion

TACE and systemic chemotherapy remain to be major treatments for most patients with advanced HCC. However, chemoresistance remains a prominent obstacle for effective treatment of HCC. Although oxaliplatin is widely used in the treatment of advanced HCC, the long-term therapeutic outcome is far from satisfaction, because most patients ultimately developed drug resistance. The mechanisms underlying chemoresistance for oxaliplatin have not been completely elucidated. In the present study, we found that ID1 was up-regulated in oxaliplatin-resistant cell lines, and that ID1 regulated hepatocellular cell malignant proliferation, apoptosis and induced oxaliplatin resistance in vitro and vivo. Further molecular mechanism revealed that ID1 conferred chemoresistance to oxaliplatin in HCC by targeting the Wnt/β-catenin pathway, regulating the expression of c-MYC, and activating the PPP. In addition, the ID1/G6PD pathway could predict poor clinical outcomes in HCC patients.

ID1 has been suggested as a potential oncogene, because it was found to be up-regulated in many types of human cancer such as breast [20], pancreas [22], kidney [23], and prostate cancers [24]. Increasing evidence indicates that ID1 is involved in many malignant biological phenotypes such as cell proliferation, immortalization, invasion, and metastasis in different types of human cancer [25, 26]. Notably, ID1 is involved in chemotherapy and radiotherapy resistance in human cancers including pancreatic, breast, lung, colorectal, and esophageal cancers [27]. For instance, in esophageal cancer, overexpression of ID1 enhanced cell resistance to etoposide-induced apoptosis, and knockdown of ID1 increased the percentage of apoptotic esophageal cancer cells. In NSCLC, silencing of ID1 in radio/chemotherapy-resistant adenocarcinoma cells sensitized adenocarcinoma cells to radiotherapy and chemotherapy [28]. Consistent with previous studies, we found in the present study that ID1 expression not only encouraged HCC cell malignant proliferation but introduced chemoresistance to oxaliplatin-induced cell apoptosis.

Metabolic reprogramming is one of the central hallmarks of cancer [21]. It has been well characterized that cancer cells typically exhibit a metabolic phenotype distinct from that of normal cells [29]. Most cancer cell types undergo metabolic reprogramming to fulfill requirements for rapid cell proliferation and also facilitate resistance to chemotherapy [30–32].Mechanistically, oxaliplatin can induce intra- and inter-strand DNA cross-links and generate formation of reactive oxygen species, which cause DNA damage and induce cell apoptosis [33]. The PPP produces two substrates, ribose-5-phosphate(R5P) and NADPH.NADPH functions as an anti-oxidant buffer to prevent ROS-induced “programmed cell death” or apoptosis [34]. R5P is important nucleotide precursor for DNA biosynthesis during the process of cell proliferation.Since ribose in nucleic acid and intracellular NADPH mainly originates from the PPP, [35] the PPP and its rate-limiting enzyme G6PD have been proposed as common targets for anticancer therapies [36]. It was found in our study that ID1-induced PPP activation was involved in HCC proliferation and oxaliplatin resistance, which is consistent with these previous findings. We observed significant ID1 expression in oxaliplaitin-resistant HCC cell lines (Fig. 1a). In addition, oxaliplaitin-resistant cell lines 97H–OXA and 3B–OXA exhibited a higher activity of G6PD with a marked increase in NADPH level (Fig. 3d and e). Conversely, inhibition of ID1 in oxaliplaitin-resistant cells suppressed G6PD expression and dampened NADPH production(Fig. 3e), which in turn augmented ROS level (Fig. 3f) and sensitized oxaliplaitin-resistant cells to death in vitro (Fig. 1e and f) and vivo (Fig. 2d). Further gain-of-function and loss-of-function studies (Fig. 4) indicated that the effect of ID1 on cell proliferation and oxaliplatin chemoresistance was mediated by G6PD activation in HCC cells. Collectively, our findings support the notion that in HCC cells, ID1 regulates the PPP, promotes cell proliferation, and confers chemoresistance to oxaliplatin by activating G6PD.

A number of oncogenes or tumor suppressors have been found to be associated with the regulation of tumor metabolism, as part of their mode of action. The oncogene c-MYC, for example, plays vital roles in cancer cell metabolic adaptation by directly regulating various genes that participate in aerobic glycolysis and glutaminolysis [37]. Apart from the expression of its target genes essential for cell cycle progression, c-MYC stimulates cancer growth by re-engineering the metabolic system. Osthus et al [38] proposed that c-MYC upregulated genes encoding glucose transporters and hexokinase to increase glucose import. Huang et al. [39] reported that c-MYC modulated glucose metabolism via regulation of miR-184/PKM2 pathway in clear-cell renal carcinoma. He et al. [40] reported the c-MYC–LDHA axis positively regulated aerobic glycolysis and promoted tumor progression in pancreatic cancer. Here, we uncovered a novel mechanism in which ID1-induced c-MYC could directly target G6PD promoter, promote G6PD transcription and activate the PPP. Interestingly, ID1 is a negatively regulatory protein. Theoretically, it is possible for ID1 to directly interact with c-MYC and negatively control G6PD transcription.To clarify the regulatory mechanism between ID1 and c-MYC, we further performed co-immunoprecipitation assay.The result showed that ID1 was able to bind to c-MYC protein and possibly inhibit G6PD transcription through protein-protein interaction.However,G6PD transcription activity assay (Fig. 6d) indicated that G6PD transcription activity was enhanced upon ID1 plasmid expression increasing. The binding of ID1 and c-MYC seemed to have minor negative impact on G6PD transcription.We attribute this positive regulatory effect to ID1-dependant Wnt/β-catenin pathway activation, which directly augments c-MYC expression (Fig. 6e), facilitates more c-MYC protein binding to G6PD promoter, thus enhances G6PD transcription.In fact, the positive regulatory effects of ID1 have been reported in previous studies.In prostate cancer, Zhang et al. [41] described that the binding of ID1 with Cav-1 could activate of Akt pathway and induce Epithelial-Mesenchymal Transition. Romero-Lanman EE [42] found ID1 maintained Embryonic Stem Cell self-renewal through up-regulating Nanog expression. Our study presented another positive regulatory role of ID1 on c-MYC/G6PD pathway activation.

## Conclusions

In summary, we described a molecular mechanism whereby ID1 regulated Wnt/ β-catenin/c-MYC pathway, which in turn activated the PPP, thus encouraging tumor proliferation and oxaliplatin chemoresistance in HCC. More importantly, ID1/G6PD signaling was found to be a predictor of unfavourable clinical prognosis in HCC patients, suggesting that the ID1 signaling pathway may be a potential therapeutic target for inhibiting HCC progression and anti-cancer drug resistance.

## Additional files

Additional file 1: Figure S1. IC50 evaluation on HCC oxaliplatin resistant cells and their parental cells. Dose-response curves between the oxaliplatin concentration and the percentage of cell activity were plotted. The data represent the mean value ± standard deviation of three independent experiments performed in triplicate. The IC50 value was 10.3 ± 0.75 for 97H vs. 1.32 ± 0.03 uM for 97H–OXA (*P* < 0.001). The IC50 value was 11.72 ± 0.83 uM for Hep3B vs. 1.31 ± 0.25 uM for 3B–OXA (*P* < 0.001). (PDF 606 kb)

Additional file 2: Table S1. Human primers used for real-time qPCR. (DOCX 12 kb)

Additional file 3: Table S2. bHLH transcription factors that could possibly bind G6PD promoter by using bioinformatic analysis. (DOCX 30 kb)

Additional file 4: Figure S2. ID1/G6PD signaling predicts unfavourable clinical prognosis in HCC patients. Differences in overall survival according to the expression of ID1 (A), G6PD (B) and their combination (C) were found to be statistically significant in HCC TCGA database. (PDF 1126 kb)

## Abbreviations

5-FU: 5-fluorouracil
6PG: 6-phosphogluconate
6PGD: 6-phosphogluconate dehydrogenase
DMEM: Dulbecco’s modified Eagle’s medium
G6P: glucose-6-phosphate
G6PD: Glucose-6-phosphate dehydrogenase
HCC: Hepatocellular carcinoma
HLH: helix-loop-helix
ID1: inhibitor of differentiation and DNA binding-1
IGF1: insulin-like growth factor 1
MEM: minimum essential medium
NADPH: nicotinamide adenine dinucleotide
NSCLC: non small-cell lung cancer
OS: overall survival
PPP: pentose phosphate pathway
R5P: ribose-5-phosphate
ROS: oxygen and species
TACE: Transcatheter arterial chemoembolization
TCGA: Cancer Genome Atlas
TUNEL: terminal deoxynucleotidyl transferase dUTP nick end labeling
