# Supplementary figures and images for: ID1 promotes hepatocellular carcinoma proliferation and confers chemoresistance to oxaliplatin by activating pentose phosphate pathway
Source: J Exp Clin Cancer Res. 2017 Nov 23;36:166. doi: 10.1186/s13046-017-0637-7 (PMC5701377; doi:10.1186/s13046-017-0637-7)

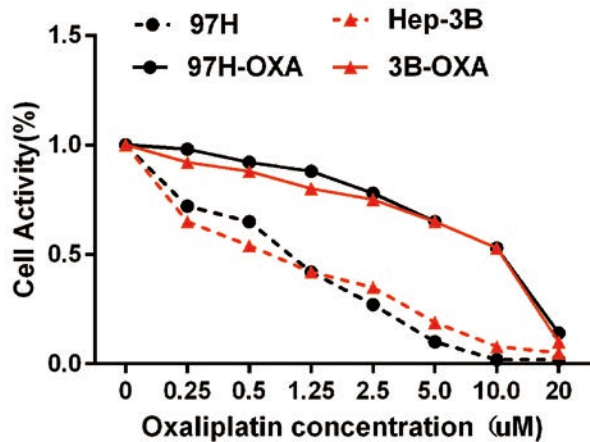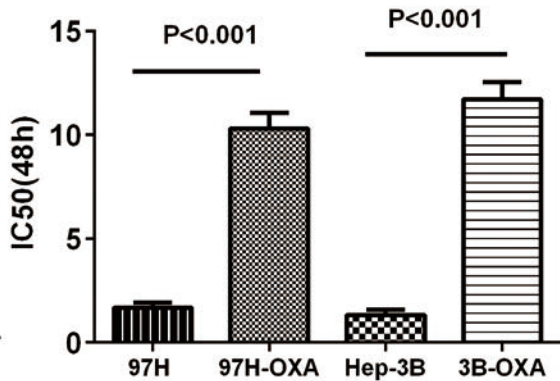

Supplement: Supplementary file 1 — IC50 evaluation on HCC oxaliplatin resistant cells and their parental cells. Dose-response curves between the oxaliplatin concentration and the percentage of cell activity were plotted. The data represent the mean value ± standard deviation of three independent experiments performed in triplicate. The IC50 value was 10.3 ± 0.75 for 97H vs. 1.32 ± 0.03 uM for 97H–OXA (P < 0.001). The IC50 value was 11.72 ± 0.83 uM for Hep3B vs. 1.31 ± 0.25 uM for 3B–OXA (P < 0.001). (PDF 606 kb) [file 13046_2017_637_MOESM1_ESM.pdf]

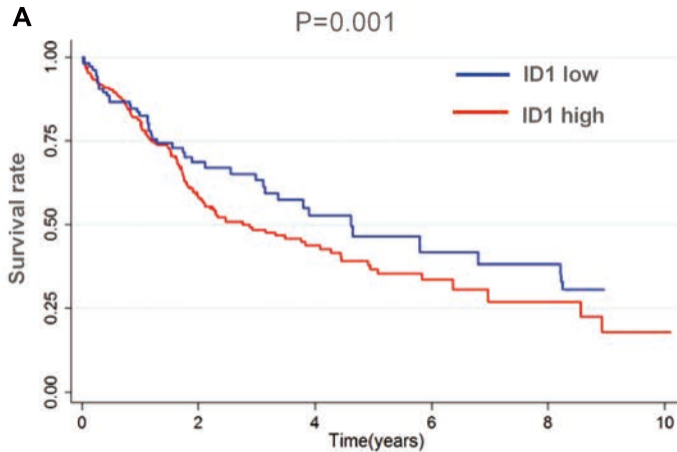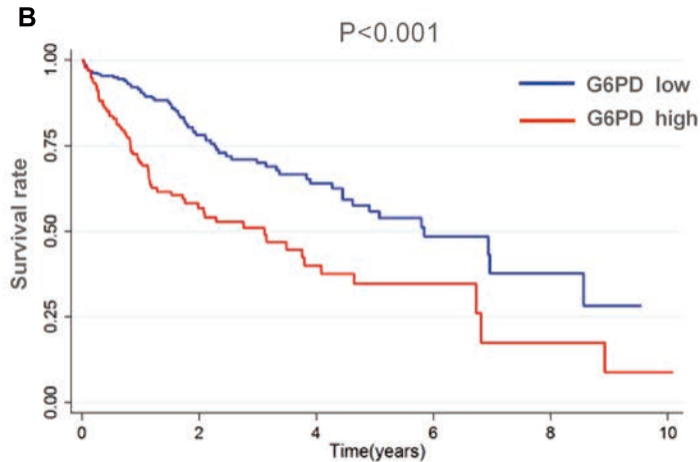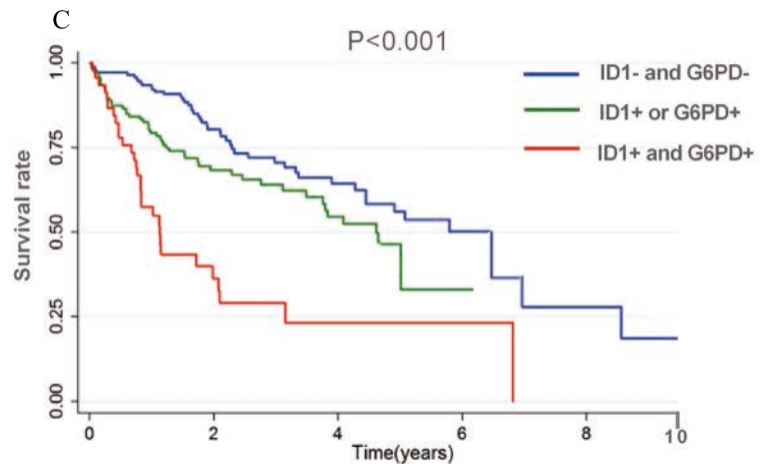

Supplement: Supplementary file 4 — ID1/G6PD signaling predicts unfavourable clinical prognosis in HCC patients. Differences in overall survival according to the expression of ID1 (A), G6PD (B) and their combination (C) were found to be statistically significant in HCC TCGA database. (PDF 1126 kb) [file 13046_2017_637_MOESM4_ESM.pdf]
