# Supplementary material for: ID1 promotes hepatocellular carcinoma proliferation and confers chemoresistance to oxaliplatin by activating pentose phosphate pathway
Source: J Exp Clin Cancer Res. 2017 Nov 23;36:166. doi: 10.1186/s13046-017-0637-7 (PMC5701377; doi:10.1186/s13046-017-0637-7)
Supplement: Supplementary file 2 — Human primers used for real-time qPCR. (DOCX 12 kb) [file 13046_2017_637_MOESM2_ESM.docx]

Supplementary Table 1 Human primers used for real-time qPCR.

| genes | Forward primer | Reverse primer |
| --- | --- | --- |
| ID1 | ACACAAGATGCGATCGTCC | GGAATCCGAAGTTGGAACC |
| c-MYC | AAACACAAACTTGAACAGCTAC | ATTTGAGGCAGTTTACATTATGG |
| G6PD | ATGGCAGAGCAGGTGGCCCT | TCATGCAGGACTCGTGAATG |
| GAPDH | AGCCACATCGCTCAGACA | GCCCAATACGACCAAATCC |
