# Supplementary material for: ID1 promotes hepatocellular carcinoma proliferation and confers chemoresistance to oxaliplatin by activating pentose phosphate pathway
Source: J Exp Clin Cancer Res. 2017 Nov 23;36:166. doi: 10.1186/s13046-017-0637-7 (PMC5701377; doi:10.1186/s13046-017-0637-7)
Supplement: Supplementary file 3 — bHLH transcription factors that could possibly bind G6PD promoter by using bioinformatic analysis. (DOCX 30 kb) [file 13046_2017_637_MOESM3_ESM.docx]

Supplementary Table 2 bHLH transcription factors that could possibly bind G6PD promoter by using bioinformatic analysis.

| Model ID | Model name | Score | Relative score | predicted site sequence |  |  |
| --- | --- | --- | --- | --- | --- | --- |
| MA0500.1 | Myog | 2.080 | 0.821891966689599 | TACAGGTGTGA |  |  |
| MA0521.1 | Tcf12 | 3.235 | 0.826046059403676 | TACAGGTGTGA |  |  |
| MA0464.1 | Bhlhe40 | 1.923 | 0.8091531526247 | CACACCTGTAA |  |  |
| MA0499.1 | Myod1 | 4.163 | 0.843728324429521 | CACACCTGTAATC |  |  |
| MA0500.1 | Myog | 1.801 | 0.818154066734597 | CACACCTGTAA |  |  |
| MA0521.1 | Tcf12 | 2.473 | 0.815407664060399 | CACACCTGTAA |  |  |
| MA0147.1 | Myc | 0.677 | 0.924344770293751 | ACACCTGTAA |  |  |
| MA0004.1 | Arnt | 6.112 | 0.830868166588692 | CAGGTG |  |  |
| MA0004.1 | Arnt | 6.112 | 0.830868166588692 | CACCTG |  |  |
| MA0499.1 | Myod1 | 2.015 | 0.816115014488121 | TCCACTTGCCATG |  |  |
| MA0147.2 | Myc | 0.678 | 0.784770267092272 | CCACTTGCCA |  |  |
| MA0104.3 | Mycn | 4.709 | 0.821707794371482 | GGCAAGTG |  |  |
| MA0500.1 | Myog | 1.358 | 0.812218978275578 | GGCAAGTGGAT |  |  |
| MA0521.1 | Tcf12 | 1.638 | 0.803750104858777 | GGCAAGTGGAT |  |  |
| MA0259.1 | HIF1A::ARNT | 4.847 | 0.810503230463245 | CCACTTGC |  |  |
| MA0147.2 | Myc | 3.276 | 0.822902390995321 | GCAAGTGGAT |  |  |
| MA0004.1 | Arnt | 6.112 | 0.830868166588692 | CACTTG |  |  |
| MA0004.1 | Arnt | 6.112 | 0.830868166588692 | CAAGTG |  |  |
| MA0104.3 | Mycn | 5.198 | 0.831100842198071 | TCCACTTG |  |  |
| MA0464.1 | Bhlhe40 | 5.064 | 0.851534919308893 | CTCAAGTGATC |  |  |
| MA0093.2 | USF1 | 8.370 | 0.883125279566128 | CTCAAGTGATC |  |  |
| MA0526.1 | USF2 | 5.506 | 0.85153874700893 | CTCAAGTGATC |  |  |
| MA0154.2 | EBF1 | 3.631 | 0.849074276758326 | ATCACTTGAGG |  |  |
| MA0595.1 | SREBF1 | 6.260 | 0.80634130964043 | ATCACTTGAG |  |  |
| MA0004.1 | Arnt | 6.112 | 0.830868166588692 | CAAGTG |  |  |
| MA0004.1 | Arnt | 6.112 | 0.830868166588692 | CACTTG |  |  |
| MA0092.1 | Hand1::Tcfe2a | 7.713 | 0.850829477068442 | GGTCAGGAAT |  |  |
| MA0092.1 | Hand1::Tcfe2a | 7.713 | 0.850829477068442 | GGTCTTGAAT |  |  |
| MA0526.1 | USF2 | 2.325 | 0.810210570953755 | GTCATGTTGGC |  |  |
| MA0093.2 | USF1 | 2.948 | 0.811852355029923 | CCAACATGACA |  |  |
| MA0499.1 | Myod1 | 4.517 | 0.848279121319305 | CCCAGCTATTTTT |  |  |
| MA0500.1 | Myog | 4.649 | 0.856310120755558 | AATAGCTGGGA |  |  |
| MA0521.1 | Tcf12 | 5.165 | 0.85299107648048 | AATAGCTGGGA |  |  |
| MA0006.1 | Arnt::Ahr | 5.297 | 0.817309426751426 | TGCATG |  |  |
| MA0006.1 | Arnt::Ahr | 5.297 | 0.817309426751426 | TGCCTG |  |  |
| MA0500.1 | Myog | 4.649 | 0.856310120755558 | AATAGCTGGGA |  |  |
| MA0521.1 | Tcf12 | 5.165 | 0.85299107648048 | AATAGCTGGGA |  |  |
| MA0499.1 | Myod1 | 2.993 | 0.828687555048032 | CCCAGCTATTTGG |  |  |
| MA0500.1 | Myog | 3.993 | 0.847521366739494 | GGAAGCTGAGG |  |  |
| MA0521.1 | Tcf12 | 3.483 | 0.829508424292302 | GGAAGCTGAGG |  |  |
| MA0499.1 | Myod1 | 3.709 | 0.837891991695166 | TTCTCCTGCCTCA |  |  |
| MA0259.1 | HIF1A::ARNT | 5.153 | 0.819631958464181 | GATCGTGC |  |  |
| MA0464.1 | Bhlhe40 | 2.383 | 0.815359969776794 | GGCTCATGCCT |  |  |
| MA0006.1 | Arnt::Ahr | 5.297 | 0.817309426751426 | TGCCTG |  |  |
| MA0259.1 | HIF1A::ARNT | 8.295 | 0.913365498918246 | GGGCGTGG |  |  |
| MA0006.1 | Arnt::Ahr | 7.680 | 0.918417219310491 | GGCGTG |  |  |
| MA0499.1 | Myod1 | 1.022 | 0.803349643551523 | TACAGTTGTGCAC |  |  |
| MA0500.1 | Myog | 2.681 | 0.829943858707366 | CACAACTGTAG |  |  |
| MA0521.1 | Tcf12 | 3.582 | 0.830890578018003 | CACAACTGTAG |  |  |
| MA0524.1 | TFAP2C | 7.278 | 0.855679327960061 | CTCAGCCGCTGGGAC |  |  |
| MA0154.2 | EBF1 | 2.173 | 0.832493559519916 | AGCCGCTGGGA |  |  |
| MA0500.1 | Myog | 4.017 | 0.847842906520569 | AGCCGCTGGGA |  |  |
| MA0521.1 | Tcf12 | 4.012 | 0.83689387197812 | AGCCGCTGGGA |  |  |
| MA0499.1 | Myod1 | 1.334 | 0.807360515386587 | CTCAGCCGCTGGG |  |  |
| MA0048.1 | NHLH1 | 8.865 | 0.821227163068019 | CCTCAGCCGCTG |  |  |
| MA0500.1 | Myog | 3.852 | 0.845632320525675 | AGCGGCTGAGG |  |  |
| MA0521.1 | Tcf12 | 3.750 | 0.833236051007072 | AGCGGCTGAGG |  |  |
| MA0524.1 | TFAP2C | 3.148 | 0.803848953430941 | TTCTGCCTCAGCCGC |  |  |
| MA0006.1 | Arnt::Ahr | 5.752 | 0.836614523526237 | CGCTTG |  |  |
| MA0092.1 | Hand1::Tcfe2a | 6.782 | 0.82339986350963 | CTCCTGGATT |  |  |
| MA0461.1 | Atoh1 | 4.461 | 0.854566543722862 | CAGGAGGC |  |  |
| MA0006.1 | Arnt::Ahr | 5.297 | 0.817309426751426 | TGGGTG |  |  |
| MA0595.1 | SREBF1 | 12.743 | 0.948699689258256 | ATCACACCAC |  |  |
| MA0596.1 | SREBF2 | 13.039 | 0.946893010841621 | GTGGTGTGAT |  |  |
| MA0461.1 | Atoh1 | 3.311 | 0.837093765427509 | CACCAGGC |  |  |
| MA0093.2 | USF1 | 5.633 | 0.84714704377572 | GCCTGGTGACA |  |  |
| MA0526.1 | USF2 | 3.621 | 0.827048457205722 | GCCTGGTGACA |  |  |
| MA0006.1 | Arnt::Ahr | 5.239 | 0.814848557272439 | TGCGAG |  |  |
| MA0461.1 | Atoh1 | 2.904 | 0.83090992128298 | CAGAAGTT |  |  |
| MA0499.1 | Myod1 | 1.527 | 0.809841599566611 | CACAGCTACCAAC |  |  |
| MA0500.1 | Myog | 3.290 | 0.838102930652157 | CACAGCTACCA |  |  |
| MA0521.1 | Tcf12 | 3.483 | 0.829508424292302 | CACAGCTACCA |  |  |
| MA0499.1 | Myod1 | 1.675 | 0.811744192616577 | GGTAGCTGTGAAT |  |  |
| MA0500.1 | Myog | 2.700 | 0.83019841103405 | GGTAGCTGTGA |  |  |
| MA0521.1 | Tcf12 | 2.579 | 0.816887545827312 | GGTAGCTGTGA |  |  |
| MA0499.1 | Myod1 | 1.810 | 0.813479666006748 | TACAGGTGTGAGC |  |  |
| MA0500.1 | Myog | 2.080 | 0.821891966689599 | TACAGGTGTGA |  |  |
| MA0521.1 | Tcf12 | 3.235 | 0.826046059403676 | TACAGGTGTGA |  |  |
| MA0464.1 | Bhlhe40 | 1.923 | 0.8091531526247 | CACACCTGTAA |  |  |
| MA0499.1 | Myod1 | 4.163 | 0.843728324429521 | CACACCTGTAATC |  |  |
| MA0500.1 | Myog | 1.801 | 0.818154066734597 | CACACCTGTAA |  |  |
| MA0521.1 | Tcf12 | 2.473 | 0.815407664060399 | CACACCTGTAA |  |  |
| MA0147.2 | Myc | 0.677 | 0.924344770293751 | ACACCTGTAA |  |  |
| MA0004.1 | Arnt | 6.112 | 0.830868166588692 | CAGGTG |  |  |
| MA0004.1 | Arnt | 6.112 | 0.830868166588692 | CACCTG |  |  |
| MA0524.1 | TFAP2C | 3.024 | 0.802292787222319 | GCCTGCCTCAGACTC |  |  |
| MA0140.2 | TAL1::GATA1 | 6.657 | 0.808215950864039 | GTGATCTGCCTGCCTCAG |  |  |
| MA0006.1 | Arnt::Ahr | 5.297 | 0.817309426751426 | TGCCTG |  |  |
| MA0092.1 | Hand1::Tcfe2a | 7.339 | 0.839810491600133 | GATCTGCCTG |  |  |
| MA0464.1 | Bhlhe40 | 5.064 | 0.851534919308893 | CTCAGGTGATC |  |  |
| MA0500.1 | Myog | 0.698 | 0.803376634296001 | CTCAGGTGATC |  |  |
| MA0093.2 | USF1 | 10.500 | 0.911124418124246 | CTCAGGTGATC |  |  |
| MA0526.1 | USF2 | 11.080 | 0.923957248527808 | CTCAGGTGATC |  |  |
| MA0464.1 | Bhlhe40 | 4.097 | 0.838487110208729 | ATCACCTGAGG |  |  |
| MA0154.2 | EBF1 | 6.855 | 0.885738359321177 | ATCACCTGAGG |  |  |
| MA0499.1 | Myod1 | 1.423 | 0.808504642288256 | ATCACCTGAGGTC |  |  |
| MA0595.1 | SREBF1 | 6.733 | 0.816727784044137 | CTCAGGTGAT |  |  |
| MA0595.1 | SREBF1 | 10.040 | 0.889345269991407 | ATCACCTGAG |  |  |
| MA0596.1 | SREBF2 | 9.048 | 0.853686750252625 | CTCAGGTGAT |  |  |
| MA0524.1 | TFAP2C | 3.288 | 0.805605915279386 | CCTGACCTCAGGTGA |  |  |
| MA0147.2 | Myc | 0.678 | 0.784770267092272 | TCACCTGAGG |  |  |
| MA0004.1 | Arnt | 6.112 | 0.830868166588692 | CAGGTG |  |  |
| MA0004.1 | Arnt | 6.112 | 0.830868166588692 | CACCTG |  |  |
| MA0595.1 | SREBF1 | 6.015 | 0.800961423321385 | CTGACCTCAG |  |  |
| MA0093.2 | USF1 | 4.055 | 0.82640401999886 | AACTCCTGACC |  |  |
| MA0092.1 | Hand1::Tcfe2a | 6.015 | 0.8008021045412 | GGTCTTGAAC |  |  |
| MA0092.1 | Hand1::Tcfe2a | 6.729 | 0.821838349526153 | AGTCTGGTCT |  |  |
| MA0091.1 | TAL1::TCF3 | 9.024 | 0.841504969127013 | CCAACATATAGT |  |  |
| MA0091.1 | TAL1::TCF3 | 8.779 | 0.836153080498697 | TCACTATATGTT |  |  |
| MA0259.1 | HIF1A::ARNT | 8.740 | 0.926640936697386 | AGGCGTGC |  |  |
| MA0006.1 | Arnt::Ahr | 7.680 | 0.918417219310491 | GGCGTG |  |  |
| MA0464.1 | Bhlhe40 | 2.367 | 0.815144080484547 | CACGCCTGCAG |  |  |
| MA0006.1 | Arnt::Ahr | 4.891 | 0.800083340398517 | CGCCTG |  |  |
| MA0461.1 | Atoh1 | 3.311 | 0.837093765427509 | CTGCAGGC |  |  |
| MA0500.1 | Myog | 4.158 | 0.849731952734388 | GGTAGCTGGGA |  |  |
| MA0521.1 | Tcf12 | 3.745 | 0.83316624526335 | GGTAGCTGGGA |  |  |
| MA0499.1 | Myod1 | 2.352 | 0.820447270284327 | CCCAGCTACCTGG |  |  |
| MA0154.2 | EBF1 | 0.965 | 0.818755900792892 | GCTACCTGGGA |  |  |
| MA0500.1 | Myog | 3.993 | 0.847521366739494 | GGAAGCTGAGG |  |  |
| MA0521.1 | Tcf12 | 3.483 | 0.829508424292302 | GGAAGCTGAGG |  |  |
| MA0499.1 | Myod1 | 3.709 | 0.837891991695166 | TTCTCCTGCCTCA |  |  |
| MA0006.1 | Arnt::Ahr | 5.752 | 0.836614523526237 | CGCTTG |  |  |
| MA0524.1 | TFAP2C | 6.847 | 0.850270395412349 | TCCTCCTCCTGGGTT |  |  |
| MA0154.2 | EBF1 | 9.840 | 0.919684477947141 | TCCTCCTGGGT |  |  |
| MA0595.1 | SREBF1 | 10.068 | 0.889960114142155 | CTCAGCTCAC |  |  |
| MA0596.1 | SREBF2 | 8.112 | 0.831827301515141 | GTGAGCTGAG |  |  |
| MA0595.1 | SREBF1 | 8.862 | 0.863477898220653 | ATCATGCCAC |  |  |
| MA0596.1 | SREBF2 | 9.965 | 0.875102470864028 | GTGGCATGAT |  |  |
| MA0092.1 | Hand1::Tcfe2a | 7.635 | 0.848531399885212 | GAGCTGGATT |  |  |
| MA0595.1 | SREBF1 | 8.683 | 0.8595472873998 | ACCACGCCAC |  |  |
| MA0596.1 | SREBF2 | 8.480 | 0.840421614693981 | GTGGCGTGGT |  |  |
| MA0006.1 | Arnt::Ahr | 7.680 | 0.918417219310491 | GGCGTG |  |  |
| MA0596.1 | SREBF2 | 7.105 | 0.808309710832826 | ATGGTCTGTT |  |  |
| MA0595.1 | SREBF1 | 6.130 | 0.803486676083386 | TTCTCCCCAC |  |  |
| MA0596.1 | SREBF2 | 6.823 | 0.801723851277302 | GTGGGGAGAA |  |  |
| MA0058.2 | MAX | 1.974 | 0.8030008122266 | GATCACATTC |  |  |
| MA0093.2 | USF1 | 5.404 | 0.844136807752335 | GGAATGTGATC |  |  |
| MA0526.1 | USF2 | 4.791 | 0.842249326738748 | GGAATGTGATC |  |  |
| MA0464.1 | Bhlhe40 | 1.647 | 0.805429062333443 | ATCACATTCCT |  |  |
| MA0154.2 | EBF1 | 3.049 | 0.842455636543817 | CTCTACTGAGA |  |  |
| MA0461.1 | Atoh1 | 12.065 | 0.970099592555785 | CAGAAGGC |  |  |
| MA0006.1 | Arnt::Ahr | 5.239 | 0.814848557272439 | TGCGTA |  |  |
| MA0091.1 | TAL1::TCF3 | 9.033 | 0.841701569117441 | GTACCATCGGGT |  |  |
| MA0461.1 | Atoh1 | 4.872 | 0.860811162748419 | CCGATGGT |  |  |
| MA0500.1 | Myog | 4.390 | 0.852840170618118 | CACAGTTGGAG |  |  |
| MA0521.1 | Tcf12 | 4.642 | 0.845689395687129 | CACAGTTGGAG |  |  |
| MA0499.1 | Myod1 | 3.888 | 0.840193100856949 | TCCAACTGTGCTC |  |  |
| MA0259.1 | HIF1A::ARNT | 6.175 | 0.850120716689533 | CTCCGTGC |  |  |
| MA0006.1 | Arnt::Ahr | 5.297 | 0.817309426751426 | TCCGTG |  |  |
| MA0464.1 | Bhlhe40 | 1.400 | 0.802096271384383 | CTAATGTGCCA |  |  |
| MA0092.1 | Hand1::Tcfe2a | 6.265 | 0.808167736538733 | CTCCTGGCAC |  |  |
| MA0154.2 | EBF1 | 4.626 | 0.860389649633648 | CTCTCCTGGCA |  |  |
| MA0595.1 | SREBF1 | 7.732 | 0.838664544994038 | CTCAGATCAC |  |  |
| MA0499.1 | Myod1 | 2.753 | 0.82560226902106 | AACATCTGCCTAT |  |  |
| MA0092.1 | Hand1::Tcfe2a | 7.307 | 0.838867690704449 | CATCTGCCTA |  |  |
| MA0464.1 | Bhlhe40 | 5.261 | 0.854193056219681 | AACATCTGCCT |  |  |
| MA0058.2 | MAX | 4.798 | 0.842574943609197 | AGGCAGATGT |  |  |
| MA0147.2 | Myc | 4.161 | 0.835891971308715 | ACATCTGCCT |  |  |
| MA0500.1 | Myog | 4.292 | 0.85152721651206 | AACATCTGCCT |  |  |
| MA0521.1 | Tcf12 | 5.044 | 0.8513017774824 | AACATCTGCCT |  |  |
| MA0499.1 | Myod1 | 5.209 | 0.857175029363742 | GGCAGATGTTGTC |  |  |
| MA0500.1 | Myog | 5.356 | 0.865782146806408 | GGCAGATGTTG |  |  |
| MA0091.1 | TAL1::TCF3 | 10.108 | 0.865184345751888 | ACAACATCTGCC |  |  |
| MA0521.1 | Tcf12 | 6.798 | 0.87578963238018 | GGCAGATGTTG |  |  |
| MA0461.1 | Atoh1 | 4.872 | 0.860811162748419 | CAGATGTT |  |  |
| MA0092.1 | Hand1::Tcfe2a | 6.388 | 0.811791627481519 | TGTCTTGCAA |  |  |
| MA0006.1 | Arnt::Ahr | 5.297 | 0.817309426751426 | TGCCTG |  |  |
| MA0464.1 | Bhlhe40 | 6.502 | 0.870937969449571 | TTCATATGCCT |  |  |
| MA0464.1 | Bhlhe40 | 6.045 | 0.864771631539773 | GGCATATGAAA |  |  |
| MA0500.1 | Myog | 3.722 | 0.843890646711516 | GGCAGCTAGGT |  |  |
| MA0521.1 | Tcf12 | 3.364 | 0.827847047591711 | GGCAGCTAGGT |  |  |
| MA0499.1 | Myod1 | 1.290 | 0.806794879614975 | CCTAGCTGCCAAA |  |  |
| MA0092.1 | Hand1::Tcfe2a | 6.348 | 0.810613126361913 | AGTTTGGCAG |  |  |
| MA0595.1 | SREBF1 | 6.598 | 0.813763356888745 | CTCACACAAG |  |  |
| MA0006.1 | Arnt::Ahr | 6.158 | 0.853840609879145 | TGTGTG |  |  |
| MA0006.1 | Arnt::Ahr | 5.239 | 0.814848557272439 | TGCGGG |  |  |
| MA0461.1 | Atoh1 | 5.280 | 0.867010200613205 | CAGCTCGC |  |  |
| MA0093.2 | USF1 | 2.587 | 0.807106961124063 | CCCAGGTTACT |  |  |
| MA0526.1 | USF2 | 2.902 | 0.817707068150811 | CCCAGGTTACT |  |  |
| MA0154.2 | EBF1 | -0.314 | 0.804210813448635 | TGCTCCAGAAG |  |  |
| MA0092.1 | Hand1::Tcfe2a | 6.620 | 0.818626933975229 | CTTCTGGAGC |  |  |
| MA0524.1 | TFAP2C | 4.659 | 0.822811591666656 | TTGGGCCCCAGCCCG |  |  |
| MA0259.1 | HIF1A::ARNT | 5.814 | 0.839351204243983 | GGACGTTC |  |  |
| MA0259.1 | HIF1A::ARNT | 4.879 | 0.811457868685565 | ATCCGTGG |  |  |
| MA0006.1 | Arnt::Ahr | 5.297 | 0.817309426751426 | TCCGTG |  |  |
| MA0524.1 | TFAP2C | 3.525 | 0.808580200694253 | CCTGTCTTTGGGGAA |  |  |
| MA0154.2 | EBF1 | 10.084 | 0.922459303054255 | TTCCCCAAAGA |  |  |
| MA0499.1 | Myod1 | 1.957 | 0.815369403698269 | GACAGGTGTGGTC |  |  |
| MA0500.1 | Myog | 10.924 | 0.940379376015928 | GACAGGTGTGG |  |  |
| MA0521.1 | Tcf12 | 10.812 | 0.931829683440433 | GACAGGTGTGG |  |  |
| MA0147.2 | Myc | 1.040 | 0.790083519152666 | ACAGGTGTGG |  |  |
| MA0464.1 | Bhlhe40 | 1.287 | 0.800571553257891 | CACACCTGTCA |  |  |
| MA0499.1 | Myod1 | 11.189 | 0.934050072869131 | CACACCTGTCAGC |  |  |
| MA0500.1 | Myog | 1.299 | 0.811428526313767 | CACACCTGTCA |  |  |
| MA0093.2 | USF1 | 2.725 | 0.808920989819378 | CACACCTGTCA |  |  |
| MA0004.1 | Arnt | 6.112 | 0.830868166588692 | CAGGTG |  |  |
| MA0004.1 | Arnt | 6.112 | 0.830868166588692 | CACCTG |  |  |
| MA0259.1 | HIF1A::ARNT | 9.014 | 0.934815026476003 | AGACGTGA |  |  |
| MA0004.1 | Arnt | 6.112 | 0.830868166588692 | CACGTC |  |  |
| MA0004.1 | Arnt | 6.415 | 0.842957366810599 | GACGTG |  |  |
| MA0259.1 | HIF1A::ARNT | 5.950 | 0.843408416688844 | AAGCGTGG |  |  |
| MA0004.1 | Arnt | 5.362 | 0.800944403663178 | AGCGTG |  |  |
| MA0006.1 | Arnt::Ahr | 8.070 | 0.934964445117472 | AGCGTG |  |  |
| MA0500.1 | Myog | 1.753 | 0.817510987172446 | CACAGCTATGA |  |  |
| MA0521.1 | Tcf12 | 2.057 | 0.809599826182705 | CACAGCTATGA |  |  |
| MA0500.1 | Myog | 2.633 | 0.829300779145215 | CATAGCTGTGG |  |  |
| MA0521.1 | Tcf12 | 2.807 | 0.820070687741048 | CATAGCTGTGG |  |  |
